# Prognostic Signature GXYLT2 Is Correlated with Immune Infiltration in Bladder Cancer

**DOI:** 10.1155/2022/5081413

**Published:** 2022-10-10

**Authors:** Shuo Wu, Shipei Qiu, Wei Chen, Liucheng Ding, Lejun Wu

**Affiliations:** ^1^Department of Urology, The First People's Hospital of Hangzhou, Linan District, Hangzhou, China; ^2^Department of Urology, The Second Affiliated Hospital of Nanjing Medical University, Nanjing, China; ^3^Department of Urology, Nanjing First Hospital, Nanjing Medical University, Nanjing, China

## Abstract

**Background:**

GXYLT2 (glucoside xylosyltransferase 2) was known as an important gene that regulates classical Notch signaling and is involved in progression in human tumors. However, the correlation between GXYLT2 expression and bladder cancer remains unclear.

**Methods:**

GXYLT2 expression was analyzed by ONCOMINE database, GEPIA database, and TIMER database. The Cancer Genome Atlas (TCGA) was utilized to confirm relationships between GXYLT2 and molecular subtypes of BLCA (bladder cancer). We discovered prognostic value of GXYLT2 in BLCA using GEPIA, LinkedOmics database, and Kaplan-Meier Plotter database. Subsequently, correlations between GXYLT2 and tumor immune infiltration were investigated through TIMER and TISIDB website. We then downloaded data of patients with BLCA from TCGA website, to conduct functional annotations and to construct protein-protein interaction network through STRING and Enrich web servers.

**Results:**

Significant differences were observed between GXYLT2 expression of bladder cancer and normal tissues. GXYLT2 was a poor prognostic biomarker in BLCA with impact on diverse clinical characteristics. We found that GXYLT2 was closely related to tumor immune infiltrated cells and immune genes. Functional annotations indicated that GXYLT2 was linked to immune-related pathways.

**Conclusions:**

The results suggested that GXYLT2 was associated with a poor prognosis and tumor immune cell infiltration of BLCA. GXYLT2 could be a promising therapeutic target in bladder cancer.

## 1. Introduction

Bladder cancer (BLCA) is one of the most common malignant tumors of urinary system, and its incidence and mortality rates are increasing worldwide [1]. For decades, the main treatment of BLCA includes traditional surgical resection, chemotherapy, and radiotherapy. With the great advances in molecular technology, immunotherapy is gradually into public view and becomes a promising treatment for BLCA [2, 3]. However, the 5-year overall survival (OS) rate is still unsatisfying, due to postoperative recurrence and distant metastasis [4, 5]. Facing these pressing challenges, it is urgent to identify novel diagnostic and prognostic targets for bladder cancer.

Immunotherapeutic strategies have achieved remarkable breakthrough in tumor treatment with providing patients unprecedented survival benefit [6]. More studies show that BLCA is identified into 2 molecular subtypes, of which 47% are luminal subtype and 35% are basal subtype [7, 8]. Tumors with different subtypes express respective gene markers and various response to immunotherapy [9], and luminal tumors tend to be poorly immune infiltrated and respond to immune checkpoint blockers [10, 11]. Distinct molecular sub-type-specific immunocompetent was one of the characterizations of tumor immune infiltrated cells (TICs), which constitute the tumor microenvironment [12, 13]. Patients with BLCA receiving immunotherapy have a benefit rate of less than 20% because of primary or secondary resistance mechanisms [14]. Therefore, it is important to identify novel immune-related therapeutic targets and provide personalized medicine for patients.

Glucoside xylosyltransferase 2 (GXYLT2) is a member of human glycosyltransferase 8 family, which has long been thought to be regulator of EGF xylosylation in Notch signaling [15]. Previous study revealed that GXYLT2 promoted human cancer cells growth, migration, and invasion activity [16]. Further research suggested that GXYLT2 was a reliable prognostic marker and correlated with tumor-infiltrating immune cells in gastric cancer [17]. These indicated that GXYLT2 may play a vital role in human tumor progression. At present, the study focused on the GXYLT2 prognosis value, and relevance for immune infiltration in BLCA remains unknown. We here analyzed the association between GXYLT2 expression and clinical characteristics or immune infiltration of BLCA by using bioinformatics databases.

## 2. Materials and Methods

### 2.1. ONCOMINE Database

ONCOMINE database (https://www.oncomine.org/) is the largest online cancer microarray database and involves 715 datasets and 86733 samples in the current edition [18]. We select ONCOMINE database to discover GXYLT2 gene expression in diverse cancer types. The mRNA expression of GXYLT2 in clinical cancer specimens was identified compared to that in corresponding normal specimens, with fold change > 2, *p* value < 0.0001, and gene rank setting to top 10%.

### 2.2. GEPIA Database

GEPIA (http://gepia.cancer-pku.cn/) is a public cancer research website for analyzing RNA sequencing expression data of 9,736 tumors and 8,587 normal samples from the TCGA and the GTEx projects [19]. In the current study, GEPIA was utilized to confirm the significance of differential gene expression and to generate the overall survive curve in BLCA. The log-rank test and the Mantel-Cox test were used in survival analysis. The differential thresholds of fold change and *p* value were set to 2 and 0.01, respectively.

### 2.3. TIMER Database

TIMER database (https://cistrome.shinyapps.io/timer/) is a comprehensive resource for analysing immune infiltrates in multiple cancer types, which includes 10897 samples and 32 cancer types from TCGA [20]. The TIMER database estimates tumor infiltrates by determination the abundances of six immune infiltrates (B cells, CD4+ T cells, CD8+ T cells, neutrophils, macrophages, and dendritic cells) [21]. This web applied modules to perform various analysis. We employed TIMER database to assess GXYLT2 expression in types of cancers and to explore the association between GXYLT2 and immune cell in filtrates levels in BLCA. Furthermore, we also analyzed the correlation between GXYLT2 and the candidate biomarker genes of tumor-infiltrated immune cells. In this study, the expression of genes in TIMER was displayed by log2 RSEM. Spearman's method was utilized to test correlation coefficient.

### 2.4. TISIDB Database

TISIDB is an integrated website portal for analyzing immune system interactions (http://cis.hku.hk/TISIDB/index.php) [22]. In this study, we adopted TISIDB to examine the correlations between GXYLT2 and immunomodulatory factors. Spearman's test was used to measure statistical significance.

### 2.5. LinkedOmics Database

The LinkedOmics database [23] (http://www.linkedomics.org/login.php) is a web-based platform for analyzing 32 TCGA cancer-associated multidimensional datasets. LinkedOmics performs survival analysis to verify prognostic value of GXYLT2 in BLCA.

### 2.6. STRING Database

In order to predict the GXYLT2-interacting candidate proteins, protein-protein interaction (PPI) analysis was performed using STRING database (https://string-db.org/)). GXYLT2 (protein name) and Homo sapiens (organism) were chosen. The PPI score of 0.4 was considered to be the cutoff for analysis.

### 2.7. Kaplan-Meier Plotter Database

Kaplan-Meier plotter (http://kmplot.com/analysis/) [23] is an online database containing microarray gene expression data and survival information derived from Gene Expression Omnibus and TCGA. It was widely used to assess the effect of 54000 genes on survival of more than 20 types of cancers. We therefore employed this database to explore the prognostic value of GXYLT2 in the patients with BLCA. In this study, we also evaluated the OS in subgroups of patients with BLCA based on clinical stage or patient gender, respectively. The Kaplan-Meier survival plot was generated with hazard ratio (HR) with 95% confidence intervals and log-rank *p* value.

### 2.8. UALCAN Database

UALCAN database (http://ualcan.path.uab.edu/) was newly developed online analysis platforms to analyze the correlation between gene transcriptional level and biomedical characteristics of cancers [24]. By utilizing this web platform, we investigated the expression of GXYLT2 in various molecular subtypes of BLCA including neuronal, basal, luminal, and luminal infiltrated luminal papillary.

### 2.9. ESTIMATE Algorithm

ESTIMATE algorithm provides a method to calculate stromal and immune scores of infiltrating stromal and immune cells through single-sample gene set enrichment analysis. We downloaded gene expression data of patients with BLCA from TCGA website (https://portal.gdc.cancer.gov), and then, raw data were normalized by log2 (*x* + 1) transformed RSEM. All data were processed in R-studio software (v3.5.3), and the “ESTIMATE” R package was used to calculate stromal and immune scores of individual patients with BLCA [25].

### 2.10. Statistical Analysis

The statistical methods used in the website tools are described above. In addition, univariate logistic regression analysis was performed to verify the relationship between GXYLT2 expression and clinicopathological characteristics in BLCA patients. All analyses were conducted in SPSS software 22.0, and *p* < 0.05 was considered statistically significant.

## 3. Results

### 3.1. The mRNA Expression Levels of GXYLT2 in BLCA

**Figure 1:**
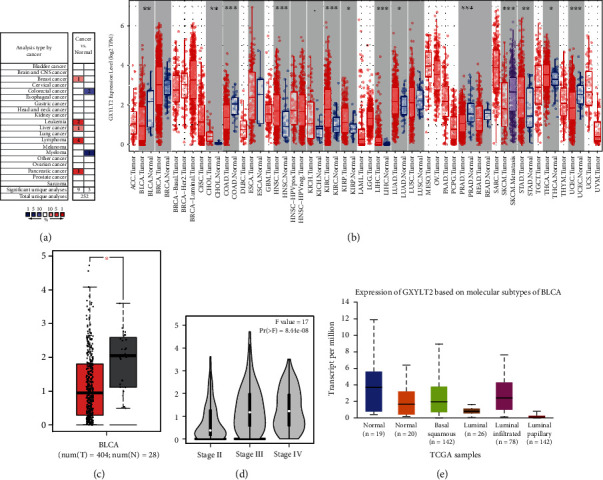
GXYLT2 mRNA expression in different human tumors including bladder cancer. (a) The expression mode of GXYLT2 in different cancers compared with normal tissues in the ONCOMINE database. (b) GXYLT2 expression levels in different cancers from TCGA database by TIMER website. (c) Decreased GXYLT2 was validated in BLCA by GEPIA website. (d) The expression of GXYLT2 increases with clinical stage in BLCA. (e) GXYLT2 expression distributes variously in different subtypes of BLCA. ^∗^*p* < 0.05, ^∗∗^*p* < 0.01, and ^∗∗∗^*p* < 0.001.

We firstly examined the expression of GXYLT2 across different human cancers by using ONCOMINE database. As shown in Figure 1(a), the expression levels of GXYLT2 were divergent in various cancers. Although GXYLT2 was in top 10% differential genes, there exist both up- and downregulated fold changes in different tumors compared to adjacent tissues. Then, we detected the expression of GXYLT2 in tumors from TCGA by utilizing TIMER database. As shown in Figure 1, the mRNA expression of GXYLT2 was significantly increased in several common cancer datasets including head-neck squamous cell carcinoma (HNSC) and kidney renal clear carcinoma (KIRC), while significant downregulation of GXYLT2 was exposed in prostate adenocarcinoma (PRAD), uterine corpus endometrial carcinoma (UCEC), colon adenocarcinoma (COAD), and bladder urothelial carcinoma (BLCA)(Figures 1(a) and 1(b)). We further used GEPIA database to validate the lower expression of GXYLT2 in BLCA (Figure 1(c)). In addition, we investigated the correlation between GXYLT2 and the stage or molecular subtypes of BLCA. The results from GEPIA database revealed a significant difference of GXYLT2 among various pathological stages of BLCA, and downregulation of GXYLT2 was observed in stage II rather than other stages (Figure 1(d)). The data from UALCAN database showed that the expression of GXYLT2 in noninfiltrated luminal subtype varied from other molecular subtypes of BLCA (Figure 1(e)). Taken together, we found that GXYLT2 was significantly reduced in BLCA compared with the adjacent normal tissues and associated with various subtypes of BLCA.

### 3.2. Association between GXYLT2 Expression and Clinicopathologic Variables in BLCA

Next, we analyzed clinical information and gene expression profile from TCGA-BLCA project. As presented in Table 1, upregulation of GXYLT2 was observed to be closely related with advanced age (≥60 vs. <60, *p* = 0.000003), while the sex of the patients was not observed to be related to gene expression. We subsequently performed subgroup analysis by TNM pathologic classification, histological subtype, tumor grade, stromal score, and immune score. The results showed significant associations between GXYLT2 expression and lymph node metastasis (*p* = 0.002) rather than distant metastasis (*p* = 0.233). Of note, higher expression of GXYLT2 was observed in advanced stages and nonpapillary group (*p* < 0.001). We also noticed that the mRNA expression of GXYLT2 was significantly associated with immune score of bladder tumor (*p* < 0.001). Since these clinical features of bladder tumor are independent prognostic factors, we hypothesized that GXYLT2 may play a role in the outcomes of patients with BLCA.

### 3.3. GXYLT2 Affects the Prognosis of Patients with BLCA

**Figure 2:**
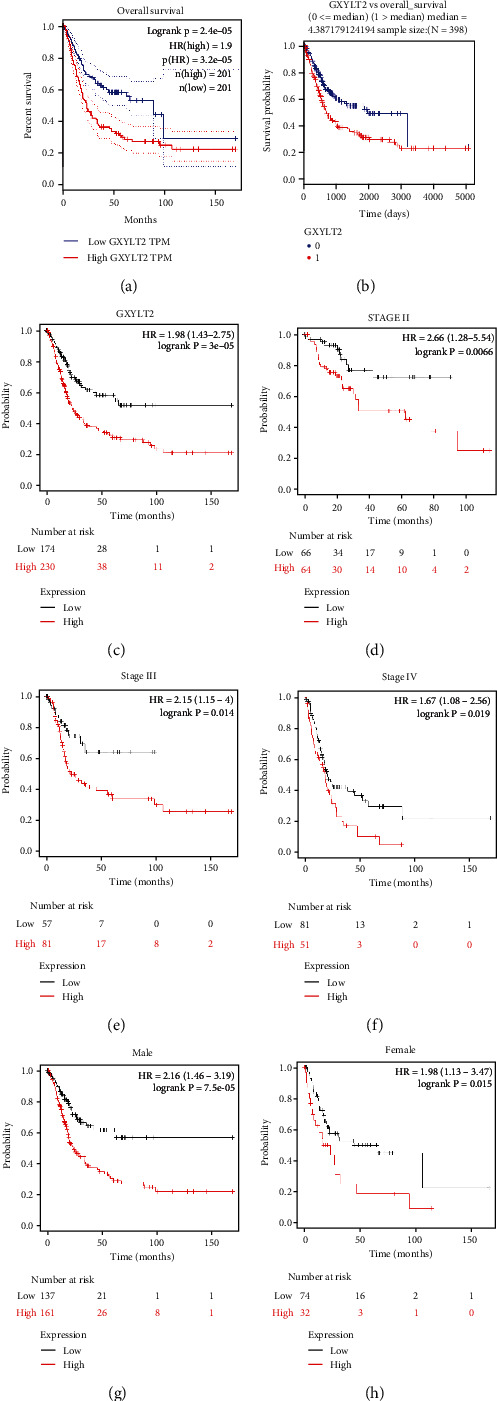
Association between GXYLT2 expression levels and the prognosis of patients with BLCA. (a) High GXYLT2 expression predicted worse OS in patients with BLCA by GEPIA website tool. (b) According to the median GXYLT2 expression, patients with bladder cancer were divided into two groups on average. High GXYLT2 expression was correlated with poor OS in the bladder cancer cohort in the LinkedOmics database. (c) OS curve in the Kaplan-Meier plotter database reflected undesirable prognosis value in BLCA. (d–f) In separate stages of BLCA subgroups, high GXYLT2 expression exhibited a poor OS rate regardless of clinical stage. (g, h) The prognostic value of GXYLT2 was independent of gender in BLCA.

To further understand the link underlying GXYLT2 and cancer patient outcome, we conducted survival analysis based on the Kaplan-Meier Plotter database, LinkedOmics database, and GEPIA database, respectively. The results showed that high GXYLT2 indicated poor overall survival of patients with BLCA in all three databases (Figures 2(a)–2(c)), which corresponded with the differential expression of GXYLT2 in various stages of BLCA. Additionally, we further analyzed whether GXYLT2 was associated with overall survival regardless of gender or stage of tumor. The results revealed that GXYLT2 was closely related with overall survival independent of tumor stage (Figures 2(d)–2(f)). Also, the effect of GXYLT2 on the prognostic potential of overall survival was not disturbed by patient gender (Figures 2(g) and 2(h)). The results above suggested that GXYLT2 could be a prognostic biomarker for BLCA patients.

### 3.4. Association between GXYLT2 and the Level of Immune Infiltration in BLCA

**Figure 3:**
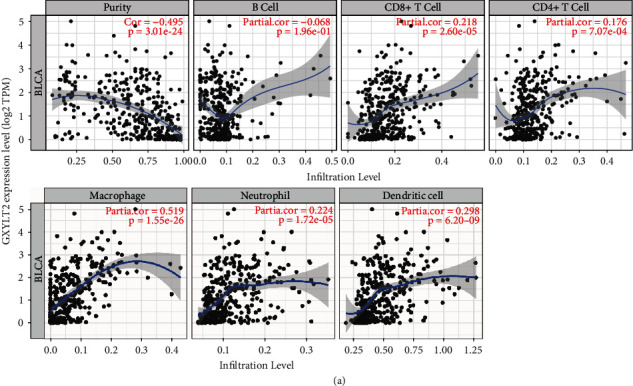
(a) Correlations between GXYLT2 expression level and BLCA immune infiltration obtained from the Tumor Immune Estimation Resource database.

By using TIMER database, we assessed the association between GXYLT2 and the levels of immune infiltration in BLCA. The proportion of tumor cells in the tumor microenvironment (TME) was known as tumor purity. As shown in Figure 3, GXYLT2 expression was significantly correlated with tumor purity as well as CD8+, macrophages, neutrophils, and dendritic cells (*p* < 0.001). Given the correlation of GXYLT2 with immune infiltration levels in BLCA, we further determined specific immune marker genes of individual immune cells. There were additional correlations between GXYLT2 and monocyte, TAM, and T cell exhaustion (Table 2). However, there was no significant association between GXYLT2 and gene markers of CD8+ T cell, T cell, B cell, M1 macrophage, and natural killer cell.

### 3.5. Correlation of GXYLT2 with Immune Markers in BLCA

**Figure 4:**
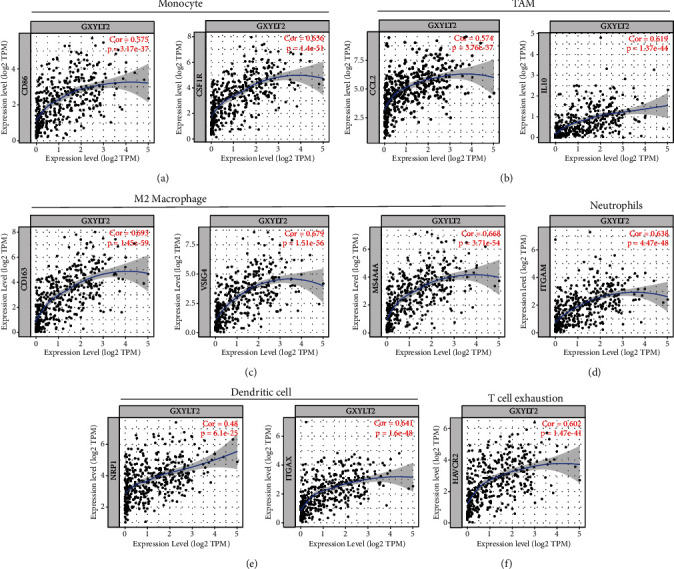
The correlation of GXYLT2 and the markers of immune effector cells. (a–f) Monocyte, TAM, M2 macrophage, neutrophils, dendritic cell, and T cell exhaustion.

We then selected outstanding gene markers of individual immune cells including monocyte, TAM, T cell exhaustion, M2 macrophages, neutrophils, and dendritic cells (correlation > 0.40, *p* < 0.001). In particular, CD86 and CSF1R (colony stimulating factor 1 receptor) of monocyte (Figure 4(a)); CCL2 (C-C motif chemokine ligand 2) and IL10 of TAM (Figure 4(b)); CD163, VSIG4 (V-set and immunoglobulin domain containing 4), and MS4A4A (membrane spanning 4-domains A4A) of M2 macrophages (Figure 4(c)); ITGAM (integrin subunit alpha M) of neutrophils (Figure 4(d)); NRP1 (neuropilin 1) and ITGAX of dendritic cell (Figure 4(e)); and HAVCR2 of T cell exhaustion (Figure 4(f)) significantly correlated with GXYLT2 expression in BLCA (*p* < 0.001) (Figure 4(a)). Taken together, these results strongly identified that GXYLT2 is associated with immune cell infiltration, suggesting that GXYLT2 may play a key role in recruitment and regulation the immune cells of BLCA microenvironment.

### 3.6. Relations between Immunomodulators and GXYLT2

**Figure 5:**
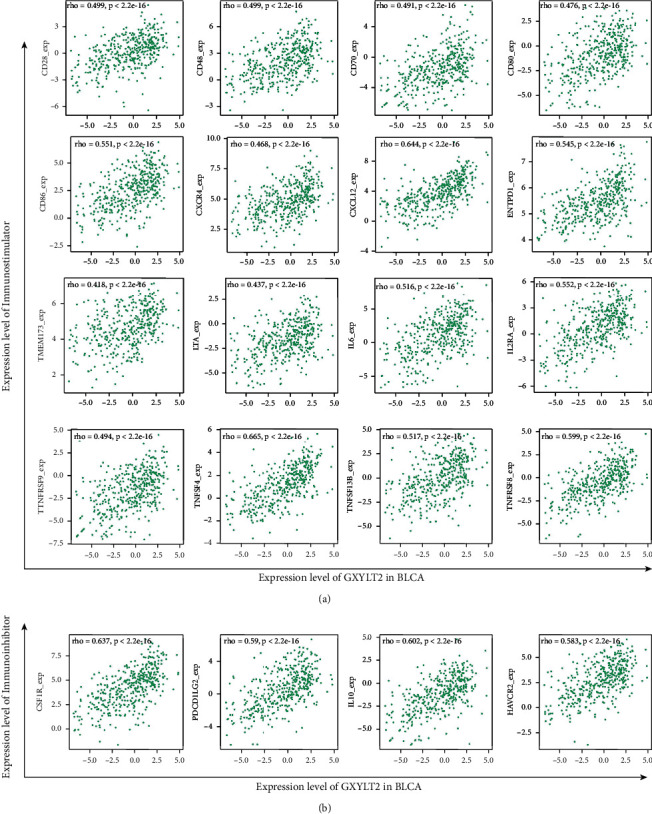
Associations of the GXYLT2 expression level with immunomodulators in BLCA. (a) Correlations between GXYLT2 expression and immunostimulators. (b) Correlations between GXYLT2 expression and immunoinhibitors.

These immunomodulators collected from Charoentong's study were an essential part in the calculation method of immunophenoscore [26], which represent the status of the immune system in solid tumors including BLCA. Immunomodulators can be divided into immunostimulators (activated CD8+ T cells and CD4+ T cells and Tem CD8+ and Tem CD4+ cells), and immunoinhibitors (Tregs and MDSCs) [27]. To further explore the relationship between GXYLT2 expression and immune molecules, we analyzed the associations between GXYLT2 and immunomodulators from TISIDB database. In detail, we found that GXYLT2 was most positively correlated with immunostimulators (CD28, CD48, CD70, CD80, CD86, CXCR4, CXCL12, ENTPD1, TMEM173, LTA, IL6, IL2RA, TNFRSF9, TNFSF4, TNFSF13B, and TNFRSF8) (Figure 5(a)) and immunoinhibitors (CSF1R, PDCD1LG2, IL10, and HAVCR2) (with rho > 0.40, *p* value < 0.05) (Figure 5(b)). It was verified again that GXYLT2 participates in modulating TME of BLCA, indicating that immunomodulator synergy could be a mechanism of GXYLT2 involving immune infiltration.

### 3.7. Enrichment Analysis of Coexpression Network of GXYLT2 and PPI Analysis in BLCA

**Figure 6:**
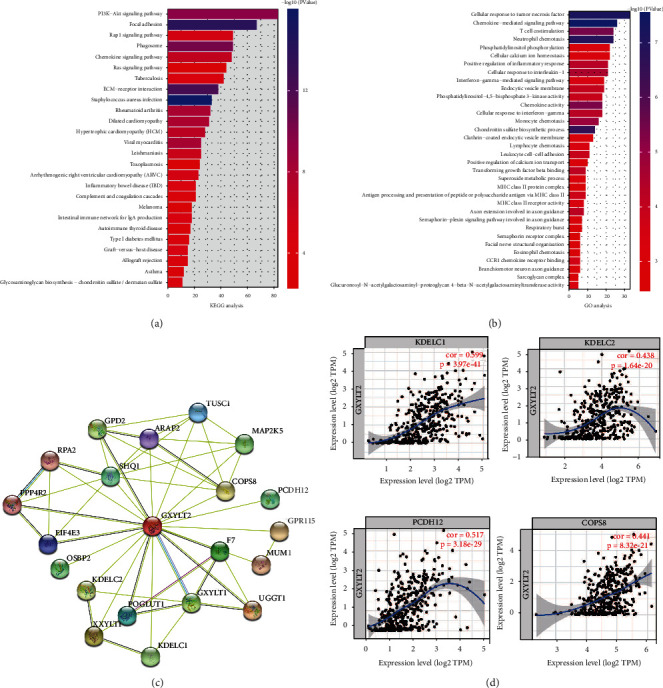
Enrichment analysis of coexpression network of GXYLT2 and PPI analysis in BLCA. (a) 26 top KEGG pathways were enriched by coexpression network of GXYLT2. (b) 34 top GO terms were enriched by coexpression network of GXYLT2. (c) 20 proteins from PPI network by the STRING database. (d) Four hub genes were further evaluated by TIMER database.

To investigate the underlying biological significance of GXYLT2 in BLCA, we finally constructed enrichment of GO and KEGG pathways by DAVID webtool 6.8. From TCGA database, we screened 1863 pathways positively correlated with GXYLT2 and 233 pathways negatively correlated with GXYLT2 in BLCA. As shown in Figure 6(a), a total of 26 significant KEGG pathways were assembled including PI3K-Akt signaling pathway with the most gene count and Staphylococcus aureus infection with the largest proportion. By the way, classical signaling pathway, PI3K-Akt signaling pathway [28], and Rap1 signaling pathway [29] were enriched in biofunction analysis of GXYLT2. These results above indicated that GXYLT2 may play a vital role in the immune response. As shown in Figure 6(b), a total of 34 significant GO terms were enriched including cellular response to tumor necrosis factor with the most gene count and chemokine-mediated signaling pathway with the largest proportion. Of note, several famous immune-related pathways were enriched in the process, such as chemokine mediated signaling pathway, neutrophil chemotaxis, T cell costimulation, and lymphocyte chemotaxis.

We analyzed protein-protein interaction network by the STRING database; 20 proteins that have highest correlation with GXYLT2 were generated into PPI network included cancer suppressor gene RPA2 and TUSC1 (Figure 6(c)). Among them, we further evaluated 4 genes that had close relationship with GXYLT2 by TIMER database. As shown in Figure 6(d), the expression of GXYLT2 had strong correlation with KDELC1, KDELC2, PCDH12, and COPS8 in BLCA.

## 4. Discussion

Since reported in 1976 by Morales et al., intravesical immunotherapy with BCG has been gradually applied in human tumors, such as bladder cancer [30]. The improving outcomes encouraged further trials of targeted immunotherapy in human cancers. Over the development of immunotherapy, high toxicity and low specificity were always an unavoidable problem [31]. It was deeply explored that TME infrastructure, with many factors they secrete, composed a variety of immune and nonimmune cell types, which eventually drive a chronic inflammatory, immunosuppressive, and proangiogenic intratumoral environment. That explained how cancer cells are able to adapt and escape from detection and eradication by host immunosurveillance. It was believed that the numbers of biological molecules and mechanistic pathways were potentially targetable for cancer treatment. In order to eradicate cancer cells, effector immune cells must first be relieved from the complex suppressive networks and activation barriers that constitute the TME [32]. For this, it will be essential to continue expanding reliable biomarkers that indicate the type of TME present in a specific tumor [33].

The expression profile from multiple databases showed GXYLT2 was dysregulated in many human tumors, notably decreased in BLCA. It indicated that GXYLT2 may participate vital immunity mechanistic pathways in tumorigenesis as reported previously in gastric cancer [16]. In the present study, the expression of GXYLT2 varied in different pathological and clinical grades of BLCA. Increased GXYLT2 correlates with poorer prognosis of survival time in BLCA. The results of clinical characteristics analysis also showed that the grade and stage of tumor had remarkable relationship with GXYLT2 expression. Furthermore, the expression of GXYLT2 was decreased in luminal and luminal papillary subtypes of BLCA, which may lead to distinct response to immunotherapy of luminal tumors. Immune and stromal scores by ESTIMATE algorithm were demonstrated to be associated with GXYLT2 in BLCA. Tumor stromal component was involved in tumorigenesis and metastasis [34]. These results suggested that GXYLT2 can impact the prognosis and may be an important factor in BLCA.

To explore the functions and mechanism of GXYLT2, we analyzed the microenvironment surrounded by bladder cancer. A total of six types of tumor immune cells including B cell, CD8+ T cell, CD4+ T cell, macrophage, neutrophil, and dendritic cell were analyzed for the correlation with GXYLT2 expression in BLCA. Among all, macrophage was most significantly associated with GXYLT2 in BLCA. It is known that macrophage is the most important source of proinflammatory cytokines during innate immune response. Besides, macrophages are the main component of the immune infiltration system in solid tumors and have been frequently accompanied with poor prognosis [35]. GXYLT2 has positive impact on TAM in BLCA according to our study, so that GXYLT2 was considered as adverse prognostic factor. Another immune cell, dendritic cell (DC), showed positive correlation with GXYLT2 in BLCA. DC is mainly responsible for providing signals required for T cell activation, which indicates that DC plays a pivotal role in the generation of antitumor T cell response [36]. DC had recently been tried as a platform of BLCA vaccines in a study [37]. By a cutoff default, we screened out the immune cell marker genes with the highest correlation with GXYLT2 including CD86, CSF1R, CCL2, IL10, CD163, VSIG4, MS4A4A, ITGAM, NRP1, ITGAX, and HAVCR2. Previous studies validated that CCL2 [38], IL10 [39], CD163 [40], and NRP1 [41] had adverse influence on progression and prognosis of BLCA, while CSF1R antagonist had been used in treatment of urothelial bladder cancers [42]. By using TISIDB, we further analyzed the relationship between GXYLT2 and immunomodulators. The correlation of GXYLT2 and immune cells was then further confirmed. As described in the previous study [27], immunomodulators comprised one of dominating categories to calculate immunophenoscore which is associated with survival in 12 solid cancers including bladder cancer. The correlationship between GXYLT2 and immunomodulators can elucidate prediction potency of GXYLT2 in poor prognosis of BLCA. In the current study, we identified 16 immunostimulators related to expression of GXYLT2, and several of them were verified to be involved in the progression of bladder cancer in the previous studies. Of them, CXCR4 and IL6 had long been thought to be oncogenes in bladder cancer which promoted various tumor behaviors [43, 44]. We also identified 4 immunoinhibitors related to expression of GXYLT2 in BLCA. Of note, PDCD1LG2 (also known as PDL2) was found to be associated with worse prognosis of BLCA by clinical data analysis [45]. These results above indicated that GXYLT2 had close link with TME and elucidated the potential mechanisms affecting tumor prognosis. By constructing GXYLT2 coexpression network in bladder cancer from TCGA database, we then performed functional enrichment analysis to elucidate gene function. The result showed that these coexpressed genes were involved in TNF response and T cell costimulation. These are classical immune and inflammatory response signaling pathways. The hub genes of PPI network also indicated broad correlations between GXYLT2 and bladder cancer progression (such as cancer suppressor gene RPA2 and TUSC1 [46, 47]. This result advanced our understanding of biology functions of GXYLT2 in BLCA.

There are some limitations in our study. The data were collected with online public databases and the mechanism of GXYLT2 regulating the infiltration of immune cells in bladder cancer should be further examined by in vivo/in vitro experiments.

## 5. Conclusion

In conclusion, GXYLT2 may serve as favorable biomarker and imply poor prognosis and clinical stage in BLCA. Our study explored that the regulation of immune infiltration may engage GXYLT2 in progression of bladder cancer. GXYLT2 was demonstrated to be close relationship with various tumor immune cells. However, further validated experiments and clinical studies are still required.

## Figures and Tables

**Table 1:** Correlation analysis between GXYLT2 and relate genes and markers of immune cells in TIMER.

Description	Gene markers	None		Purity	
		Cor	*p*	Cor	*p*
CD8+ T cell	CD8A	0.353	∗∗∗	0.134	∗
	CD8B	0.305	∗∗∗	0.156	∗∗

T cell (general)	CD3D	0.298	∗∗∗	0.03	0.561
	CD3E	0.403	∗∗∗	0.133	∗
	CD2	0.389	∗∗∗	0.126	∗

B cell	CD19	0.381	∗∗∗	0.185	∗∗∗
	CD79A	0.405	∗∗∗	0.187	∗∗∗

Monocyte	CD86	0.575	∗∗∗	0.386	∗∗∗
	CD115 (CSF1R)	0.656	∗∗∗	0.502	∗∗∗

TAM	CCL2	0.574	∗∗∗	0.411	∗∗∗
	CD68	0.406	∗∗∗	0.228	∗∗∗
	IL10	0.619	∗∗∗	0.488	∗∗∗

M1 macrophage	INOS (NOS2)	0.174	∗∗∗	0.131	∗
	IRF5	-0.107	∗	-0.105	∗
	COX2 (PTGS2)	0.127	∗	0.031	0.549

M2 macrophage	CD163	0.693	∗∗∗	0.565	∗∗∗
	VSIG4	0.679	∗∗∗	0.537	∗∗∗
	MS4A4A	0.668	∗∗∗	0.531	∗∗∗

Neutrophils	CD66b (CEACAM8)	0.02	0.683	0.047	0.372
	CD11b (ITGAM)	0.638	∗∗∗	0.485	∗∗∗
	CCR7	0.044	0.378	-0.06	0.254

Natural killer cell	KIR2DL1	0.221	∗∗∗	0.081	0.122
	KIR2DL3	0.229	∗∗∗	0.057	0.278
	KIR2DL4	0.21	∗∗∗	0.026	0.619
	KIR3DL1	0.196	∗∗∗	0.067	0.201
	KIR3DL2	0.18	∗∗∗	0.031	0.558
	KIR3DL3	0.031	0.53	-0.014	0.794
	KIR2DS4	0.18	∗∗∗	0.04	0.439

Dendritic cell	HLA-DPB1	0.513	∗∗∗	0.309	∗∗∗
	HLA-DQB1	0.431	∗∗∗	0.225	∗∗∗
	HLA-DRA	0.451	∗∗∗	0.243	∗∗∗
	HLA-DPA1	0.469	∗∗∗	0.278	∗∗∗
	BDCA-1 (CD1C)	0.266	∗∗∗	0.082	0.117
	BDCA-4 (NRP1)	0.48	∗∗∗	0.38	∗∗∗
	CD11c (ITGAX)	0.641	∗∗∗	0.479	∗∗∗

Th1	T-bet (TBX21)	0.375	∗∗∗	0.143	∗∗
	STAT4	0.435	∗∗∗	0.206	∗∗∗
	STAT1	0.335	∗∗∗	0.156	∗∗
	IFN-*γ* (IFNG)	0.272	∗∗∗	0.089	0.0891
	TNF-*α* (TNF)	0.282	∗∗∗	0.16	∗∗

Th2	GATA3	-0.387	∗∗∗	-0.266	∗∗∗
	STAT6	-0.179	∗∗∗	-0.125	∗
	STAT5A	0.28	∗∗∗	0.122	∗
	IL13	0.185	∗∗∗	0.041	0.434

Tfh	BCL6	-0.109	∗∗∗	-0.08	0.125
	IL21	0.172	∗∗∗	0.092	0.0782

Th17	STAT3	0.393	∗∗∗	0.273	∗∗∗
	IL17A	-0.098	∗	-0.157	∗∗

Treg	FOXP3	0.446	∗∗∗	0.277	∗∗∗
	CCR8	0.45	∗∗∗	0.297	∗∗∗
	STAT5B	0.214	∗∗∗	0.226	∗∗∗
	TGF*β* (TGFB1)	0.357	∗∗∗	0.261	∗∗∗

T cell exhaustion	PD-1 (PDCD1)	0.394	∗∗∗	0.17	∗∗
	CTLA4	0.391	∗∗∗	0.168	∗∗
	LAG3	0.394	∗∗∗	0.186	∗∗∗
	TIM-3 (HAVCR2)	0.602	∗∗∗	0.42	∗∗∗
	GZMB	0.369	∗∗∗	0.118	∗

*p* < 0.05 was considered to be statistically significant (^∗^*p* < 0.05, ^∗∗^*p* < 0.01, and ^∗∗∗^*p* < 0.001).

**Table 2:** The correlation of GXYLT2 expression with the clinical features of BLCA.

Characteristics	Total	OR (95%)	*p* value
Age	407	1.311 (1.171-1.469)	0.000003
Gender	407	0.913 (0.825-1.011)	0.081
Subtype (non-p)	402	0.691 (0.621-0.769)	1.19E-11
N (0VS+)	365	1.180 (1.065-1.306)	0.002
M (0VS1)	206	1.172 (0.903-1.520)	0.233
Stromal	408	2.685 (2.240-3.220)	1.48E-26
Immune	408	1.568 (1.403-1.753)	2.51E-15
Stage (3+4 vs. 1+2)	405	1.396 (1.258-1.550)	3.83E-10
T (3+4 vs. 1+2)	373	1.397 (1.246-1.544)	2.39E-09
Grade (high/low)	404	3.355 (2.045-5.504)	0.000002

## Data Availability

The datasets used in our study are available freely from the open databases.

## Abbreviations

BLCA: Bladder cancer
TIMER: Tumor immune estimation resource
TME: Tumor microenvironment
OS: Overall survival
CTLA4: Cytotoxic T lymphocyte associated antigen 4
TAMs: Tumor-associated macrophages
DC: Dendritic cell
Rap1: Ras oncoprotein 1
PD-1: Programmed death protein-1
PD-L1: PD ligand-1
TICs: Tumor immune infiltrated cells
ICIs: Immune checkpoint inhibitors
PPI: Protein-protein interaction
GO: Gene Ontology.
